# Birth defects in newborns and stillborns: an example of the Brazilian reality

**DOI:** 10.1186/1756-0500-4-343

**Published:** 2011-09-09

**Authors:** Camila Ive Ferreira Oliveira, Antonio Richieri-Costa, Valéria Cristina Carvalho Ferrarese, Denise Cristina Móz Vaz, Agnes Cristina Fett-Conte

**Affiliations:** 1 Departamento de Biologia, Instituto de Biociências, Letras e Ciências Exatas, Universidade Estadual Paulista - UNESP, São José do Rio Preto, SP, Brasil; 2 Hospital de Reabilitação de Anomalias Craniofaciais, Universidade de São Paulo - USP, Bauru, SP, Brasil; 3 Departamento de Biologia Molecular, Faculdade de Medicina de São José do Rio Preto, São José do Rio Preto, SP, Brasil; 4 Departamento de Ginecologia e Obstetrícia, Faculdade de Medicina de São José do Rio Preto, São José do Rio Preto, SP, Brasil

## Abstract

**Background:**

This study constitutes a clinical and genetic study of all newborn and stillborn infants with birth defects seen in a period of one year in a medical school hospital located in Brazil. The aims of this study were to estimate the incidence, causes and consequences of the defects.

**Methods:**

For all infants we carried out physical assessment, photographic records, analysis of medical records and collection of additional information with the family, besides the karyotypic analysis or molecular tests in indicated cases.

**Result:**

The incidence of birth defects was 2.8%. Among them, the etiology was identified in 73.6% (ci95%: 64.4-81.6%). Etiology involving the participation of genetic factors single or associated with environmental factors) was more frequent 94.5%, ci95%: 88.5-98.0%) than those caused exclusively by environmental factors (alcohol in and gestational diabetes mellitus). The conclusive or presumed diagnosis was possible in 85% of the cases. Among them, the isolated congenital heart disease (9.5%) and Down syndrome (9.5%) were the most common, followed by gastroschisis (8.4%), neural tube defects (7.4%) and clubfoot (5.3%). Maternal age, parental consanguinity, exposure to teratogenic agents and family susceptibility were some of the identified risk factors. The most common observed consequences were prolonged hospital stays and death.

**Conclusions:**

The current incidence of birth defects among newborns and stillbirths of in our population is similar to those obtained by other studies performed in Brazil and in other underdeveloped countries. Birth defects are one of the major causes leading to lost years of potential life. The study of birth defects in underdeveloped countries should continue. The identification of incidence, risk factors and consequences are essential for planning preventive measures and effective treatments.

## Background

Birth defects (BD) or congenital anomalies include all structural and functional alterations in embryonic or fetal development resulting from genetic, environmental or unknown causes, which result in physical and/or mental impairment. There may be single or multiple alterations with major or minor clinical significance [1,2]. The incidence of BD is 3 to 5% among newborn babies [3]. BD comprise a complex and heterogeneous group of embryonic and/or fetal development disorders, which, in about 50% of cases, have no known cause, although genetic and environmental or a combination of these two may be involved [4].

Among the risk factors are advanced maternal and paternal ages, parental consanguinity, teratogenic agents, such as infectious agents and drugs, and nutritional deficiencies [5-7].

One of the consequences of BD is the high death rate within the first year of life. Infant mortality is an important indicator of the health of a country or community and is linked to factors such as maternal health, quality of and access to health services, socio- economic conditions and public health practices [8]. In developed countries, BD are the main cause of infant mortality. A similar trend is being observed in the developing world [9]. In Brazil, BD jumped from the fifth to the second cause of deaths of under one-year-old children between 1980 and 2000, but the data is still very limited [8]. Affected children are four times more likely to die within the first year of life. Another consequence of BD is the risk of have more severe clinical complications and a higher number of hospitalizations [10-12].

Information on BD is becoming increasingly more important throughout the world in order that preventive measures can be taken. Accurate diagnoses are also a prerequisite for the prognosis and planning of the conduct with individual patients, as well as for genetic counseling of the family [13].

This article describes the findings of a prospective clinical genetic study performed of newborn and stillborn infants with BD treated in a period of one year at a teaching hospital of Brazil. The aims of the study were to evaluate the incidence, the diagnoses, causes and consequences of BD.

## Methods

This study was undertaken only after approval by the institution's Research Ethics Committee (Number 341/2008) and the National Research Ethics Committee and after obtaining written consent from parents. All newborn and stillborn babies treated in Hospital de Base (HB) in São José do Rio Preto from 1st August 2008 to July 31 2009 were studied.

HB is a medical school hospital that serves about of 50,000 patients per month in 40 specialties. Sao José do Rio Preto is located in the northwestern region of the state of Sao Paulo, Brazil. It is the regional center of 99 municipalities with a population of around one and a half million inhabitants.

The investigations were performed by a multidisciplinary team involving neonatologists and geneticists of the HB. Photographs were taken of the baby, and a detailed physical examination, including identification of the details of the birth and body measurements. Epidemiological and clinical data and the results of tests were obtained from the patients' medical records and at least one parent was interviewed to collect additional background data (previous obstetrics history, gestation information, personal and family histories). A sample of peripheral or umbilical cord blood was collected for a karyotypic study in order to confirm or investigate chromosomal abnormalities. Other different laboratory tests were performed depending on the needs of each individual.

We studied the incidence of BD, possible diagnoses and causes (genetic, environmental and unknown). Etiology involving the participation of single genetic factor was named "G", possibly genetic factors in association with environmental factor was named "G/E" and those caused exclusively by environmental factors was named "E". The death rate and time of hospitalization were the consequences investigated. Cases where there was a combination of two or more major defects for which no etiological factor was demonstrated or suspected were categorized as "multiple defects". Data were analyzed descriptively. A calculation of central tendency (mean) was performed for quantitative variables and frequencies are reported for qualitative variables.

## Results

We identified 110 individuals with BD during one year of study, 15 of them born in other hospitals and were treated in our hospital. At the HB, 3026 infants were born during the period of study and 85 of them had defects, that means an incidence of 2.8% (exact confidence interval 95%: ci95%: 2.2-3.4%) of BD. Of 110, 103 (93.6%; ci95%: 87.3-97.4%) were newborn babies and seven (6.4%; ci95%: 2.6-12.7%) were stillbirths; 61 (55.5%; ic95%: 45.7-64.9%) were male, 48 (43.6%; ci95%: 34.2-53.4%) were females (ratio of 1.3:1) and one (0.9%; ci95%: 0.0-5.0%) were of indeterminate sex. Two cases had indeterminate sex. One was elucidated after clinical and laboratory tests, including karyotyping (46, XX). The other, a stillbirth can not be elucidated.

Nineteen (17.3%; ci95%: 10.7-25.7%) cases were antenatally diagnosed with BD and the mothers preferred to continue their pregnancy.

The maternal age ranged from 13 to 46 years old (average 26 years with standard deviation of 7.1; ci95%: 24.9-27.6). The majority of mothers (64.8%; ci95%: 55.0-73.8%) were aged from 20 to 34 years, 21.3% (ci95%: 14.0-30.2%) were aged up to 19 years old, 13.8% (ci95%: 8.0-21.9%) ≥ 35 years old, and in 2% the age was unknown. The paternal age ranged from 17 to 51 years old (mean 29.2 years with standard deviation of 7.5, ci95%: 27.6-30.8). No infants of had BD associated with advanced paternal age (45 years or older). The period of gestation ranged from 25 to 41 weeks (mean = 35.4 weeks; ci95% 34.8-36.0). In respect to the gestational age, 67.0% (ci95%: 57.2-75.8%) were preterm and 33.0% (ci95%: 24.2-42.8%) were born at term. The majority of children (77.3%; ci95%: 68.3-84.7%) were delivered by cesarean section and 51.8% (ci95%: 42.1-61.4%) had low birth weight (< 2500 g). Of these, 85.7% (ci95%: 73.8-93.6%) were preterm, 5.4% (ci95%: 1.1-14.9%) had intrauterine growth restriction (IUGR) and 8.9% (ci95%: 3.0-19.6%) were underweight, were born at term and showed no IUGR.

One hundred and four (94.6%; ci95%: 88.5-98.0%) pregnancies were single gestations and six (5.5%; ci95%: 2.0-11.5%) were twins, two monozygotic twins, two dizygotic and two unconfirmed zygosity. One of the twins were monozygotic and had gastroschisis. The mother was 17 years old.

Gestational diabetes mellitus was considered as a teratogenic factor in 4% of infants. They presented heart abnormalities associated with minor BD (3) and Caudal Dysplasia Sequence (1). Two mothers (1.8%; ci95%: 0.2-6.4%) reported excessive alcohol consumption (several doses on several days of the week) during the pregnancy and their children had the phenotypes compatible with Fetal Alcohol Syndrome. Two families (1.8%; ci95%: 0.2-6.4%) reported that the child was the result of a consanguineous marriage; one child had multiple defects of unknown etiology and the parents were first cousins and another had thoracic dystrophy (Jeune's syndrome) and the parents were second cousins.

Familial recurrence of the BD occurred in six (5%; ci95%: 2.0-11.5%) families. BD were observed in one of the parents in three cases: one mother had cleft of lip and palate, one mother had dwarfism (Hypochondroplasia) and the father of other baby had polythelia. In another three cases, the defect of the newborn baby was observed in siblings: two had heart disease and one had neural tube defect.

Karyotypic alterations was detected in 13 (11.8%; ci95%: 6.4-19.4%) infants, all were numerical and involving autosomes. Among the alterations, the most common finding was trisomy 21 (69%, ci95%: 39-91%), followed by trisomy 18 (23%, ci95%:5-54%) and trisomy 13 (8%, ci95%: 0.1-36%).

The etiology was suggested in 73.6% cases. Etiology involving the participation of single genetic factors (G = 43.2%; ci95%: 32.2-54.7%) or associated with environmental factors (G/E = 49.4%, ci95%: 38.0-60.7%) was more frequent than those caused exclusively by environmental factors (alcohol in and gestational diabetes mellitus - E = 7.4% (ci95%: 2.8-15.4%). Isolated congenital heart disease and Down syndrome were the most common BD, followed by isolated gastroschisis, neural tube defects and isolated clubfoot. 62.5% of the mothers of infants with gastroschisis were aged up to 20 years old and 54% of the mothers of children with numerical chromosomal abnormalities were 35 years old or older. Table 1 presents the cases of BD with and without diagnoses that were studied.

**Table 1:** Birth defects with conclusive or presumed (*) diagnosis and etiology

Diagnosis	Number of cases		Etiology
	**Newborns**	**Stillborns**	

Multiple Defects	10	5	U
Congenital Heart Disease	9		G/E
Down Syndrome	9		G
Gastroschisis	8		G/E
Neural Tube Defects	7		G/E
Foot-clubfoot	5		G/E
Amniotic Band Sequence	4		U
Disruption by Diabetes	3		E
Edwards Syndrome	2	1	G
Orofacial Ccenter	3		G/E
Association VATERR	2		U
Esophageal Atresia	2		G/E
Fetal Alcohol Syndrome	2		E
Lung Cystic Adenomatous Malformation	2		U
Polydactyly (OMIM 603596)	2		G
Velocardiofacial Syndrome (OMIM 192430)*	2		G
Anorectal anomaly	1		G/E
Atelosteogenesis Type III (OMIM 108721)*	1		G
Biliary Atresia Extrahepatic (OMIM 210500)	1		U
Campomelic Dysplasia (OMIM 114290)	1		G
Camptodactyly (OMIM 114200)	1		G
Caudal Dysplasia Sequence	1		E
Colpocephaly		1	G/E
Congenital Aplasia Cutis (OMIM 107600)	1		G
Dandy-Walker (OMIM 22020)	1		G
Defects by Autosomal Recessive Inheritance*	1		G
DiGeorge syndrome (OMIM 188400)	1		G
Distal Arthrogryposis (OMIM 601680)	1		G
Duodenal atresia	1		U
Hypochondroplasia (OMIM 146000)	1		G
Imperforate Anus	1		G/E
Larsen Syndrome (OMIN 150250)	1		G
Laterality Sequence	1		U
Limb Body Wall Complex	1		U
Meckel Diverticulum	1		G/E
Möebius Sequence	1		U
Oculo-Auriculo-Vertebral Spectrum	1		U
Omphalocele	1		G/E
Patau Syndrome	1		G
Penoscrotal Hypospadia	1		G/E
Pfeiffer syndrome (OMIM 101600)*	1		G
Polythelia (OMIM 163700)	1		G
Prune Belly Syndrome (OMIM 100100)	1		G
Syndrome Barber-Say (OMIM 209885)*	1		G
Thanatophoric Dysplasia Type II (OMIM 187601)	1		G
Thoracic Dystrophy 1 (Jeune) (OMIM 208500)	1		G
Townes Brocks Syndrome (OMIM 107480)	1		G
X-Linked Hydrocephalus (OMIM 307000)	1		G

E = environmental.

G = genetic factors

G/E = genetic factors or in combination with environmental factors.

U = unknown.

The average hospital stay was 33 days. The longest stay, a case of gastroschisis with surgical complications, was 254 days. Death during hospitalization occurred in 35 of 103 in patients that were alive (34.0%; ci95%: 24.9-44.0%) cases and 25 of them (71.4%; ci95%: 53.7-85.4%) were neonatal (< 28 days). The most frequent cause was infection.

## Discussion

If the prevalence and etiology of BD in a specific population is not known, effective public health policies, specially related with preventive measures can not be developed. This knowledge is essential to define the existence of particular situations and to compare the characteristics and possible causal factors with other populations, which can result in the definition of strategies to minimize damages.

The frequency of BD can vary with the cultural, social, geographic and genetic origin of each population. Moreover, the methods of investigation and observation and classification criteria used differ among authors. Most studies report an incidence of between 3% and 5% of BD in different populations of the world. There are few data available for Brazil and nationally representative data are underestimated. The reported frequencies vary between 1.7 and 5% [14-16]. The incidence of 2.8% that we observed is compatible with previous reported data. Incidence by gender was similar although showed a slightly higher in men (1.3:1), what was observed previously [17].

Maternal age was a risk factor for BD in our study. This relationship is already well established. Early ages (up to 20 years) are associated with gastroschisis [18] and all the mothers that the child had this BD were aged up to 20 years old. At older ages the risk of numerical chromosomal abnormalities increases progressively [19] and this was a risk factor observed in this study too, in which most mothers of children with these abnormalities were 35 years or older. The cause of gastroschisis is unknown, although possible exogenous causes have been studied. The diagnosis of gastroschisis in twin pregnancy has been described [20,21], as occurred in one of our cases.

BD is more frequently observed in premature children and those weighing less than 2500 g and measuring less than 45 cm at birth [22]. This was also observed in our study population. The majority, about two thirds of cases, were delivered by Cesarian- section. These high numbers possibly occurred due to fetal indications because of a prenatal diagnosis of BD, such as gastroschisis, hydrocephalus and giant omphalocele and meningoceles at risk of dystocia or rupture. However, opting for cesarean is also very common in Brazil, regardless of the fetal status [23].

Gestational diabetes, alcohol consumption, parental consanguinity and others affected people in the same family were others risk factors associated with BD in our cases.

Gestational diabetes mellitus is assumed to be a risk factor in some cases as it is related to limb and heart defects [24]. Prenatal alcohol exposure is a risk factor for BD and can lead to a wide range of adverse effects on a developing fetus [25]. It has long- lasting adverse effects, causing structural, behavioral and cognitive damage [7].

Inbreeding is known to increase the risk of morbidity and mortality for the progeny [26]. Parental consanguinity was observed in approximately 2% of cases which is higher than the general population of South America (0.96%) However, this is lower than previously reported frequencies of consanguinity among parents of children with BD in Brazil of up to 8.5% [19]. This can be explained by the wide variation of consanguinity in different cities and regions of Brazil. Our study was carried out in a city in a well developed region of the country, with low rates of immigration of individuals from other less developed regions, as northeast where consanguinity is high [27].

The failure to diagnose can harm the process of genetic counseling. In this study, a conclusive or presumed diagnosis and etiology were possible in most cases, which facilitated the process of genetic counseling of families.

The observed incidence Down syndrome, defects of the neural tube, congenital club foot and amniotic rupture sequence were expected to be among the most commonly observed abnormalities in population. However, gastroschisis occurred in 2.64:1000 newborn babies, when 1:4000 was expected [18].

The hospital stay of newborn babies without BD or without intercurrences is short, from 24 (normal delivery) to 48 hours (c-section) in Brazil. The average length of hospitalization in the cases studied here was much longer, about 33 days, that is explained by the severity of BD, surgical complications and hospital infections related to them. This is one of the expected consequences of BD and generates a high onus that must be considered in health policies [11].

When the causes of infant mortality are analyzed, a decrease in the total number of deaths is observed in several regions of the world, especially deaths related to infections and nutritional deficiency. As a result, the proportion of deaths attributable to BD is increasing [8]. BD account for a high rate of early mortality and are more frequent among stillborn [16]. In developed countries BD are responsible for about 20% of neonatal deaths [28]. In a study of pediatric deaths in a children's hospital in the USA, 34.4% were due to BD [29]. In Brazil, official data are scarce, but BD account for

almost 30% of deaths in under one-year-old children in some states, such as Rio Grande do Sul. Evolution to death can occur for different secondary to BD, including prematurity or environmental causes, including complications of medical or surgical interventions and infections [30]. In this study, 6% of cases with BD were stillborn with multiple and major BD. Moreover, most of the deaths of newborns occurred in the neonatal period. Thus, the mortality rate observed here can be considered high, which is one of the consequences of BD. Isolated anomalies of the cardiovascular and urinary systems, in general, are not easily detected at birth and their diagnosis involves adequate postnatal care [30]. In this study, cardiovascular system anomalies were the most common and urinary tract defects were only observed in patients with multiple BD, perhaps as most cases are asymptomatic in the neonatal period.

## Conclusions

The current incidence of birth defects among newborns and stillbirths in our population is similar to those obtained by other studies performed in Brazil and in other underdeveloped countries. Birth defects are among the causes leading to lost years of potential life. The study of birth defects in underdeveloped countries should continue. The identification of incidence, risk factors and consequences are essential for planning preventive measures and effective treatments.

## List of abbreviations

BD: Birth defects; HB: Hospital de Base; G: Genetic factors; G/E: Genetic factors or in combination with environmental factors; U: Unknown; E: Environmental

## Competing interests

The authors declare that they have no competing interests.

## Authors' contributions

CIFO carried out the data collection, karyotypic analysis and confection of the manuscript. VCCF carried out the karyotypic analysis. ARC participated in the investigation of the diagnoses. DCMV participated in the data collection. ACF-C designed the study, clinical assessment, oversaw the whole study and did the critically reviewing manuscript. All authors read, revised and approved the final manuscript.
